# Age and gender influence on clinical manifestations of thyroid-associated ophthalmopathy: a case series of 2479 Chinese patients

**DOI:** 10.3389/fendo.2024.1434155

**Published:** 2024-10-03

**Authors:** Mei Yang, Weimin He

**Affiliations:** ^1^ Department of Ophthalmology, West China Hospital of Sichuan University, Chengdu, Sichuan, China; ^2^ Department of Ophthalmology, The 3rd Affiliated Hospital of Chengdu Medical College, Pidu District People's Hospital, Chengdu, China

**Keywords:** Thyroid-Associated Ophthalmopathy, age, gender, severity, clinical

## Abstract

**Background:**

Significant individual differences exist in the clinical manifestations of thyroid-associated ophthalmopathy (TAO). Age and gender have an impact on the presentation and severity of TAO.

**Objective:**

To evaluate the clinical characteristics of TAO patients, with particular consideration of their age and gender.

**Methods:**

Demographic and clinical data of 2479 TAO patients were collected. Patients were divided into 7 groups based on age: Group 1: ≤18 years old; Group 2: 19-29 years old; Group 3: 30-39 years old; Group 4: 40-49 years old; Group 5: 50-59 years old; Group 6: 60-69 years old; Group 7: ≥70 years old. Compared clinical manifestations among different groups and between males and females.

**Result:**

In age group 1, the ratio of female to male TAO patients was 3.79, and in age group 7, it was 0.86. As age increased, there was a decline in the percentage of females (γ=-0.168, *p*<0.001). During the aging process, the proportion of TAO patients with unilateral involvement also gradually decreased (γ=-0.23, *p*<0.001). In addition, the proportion of TAO patients with upper eyelid retraction ≥2mm and exophthalmometry ≥17mm also decreased (γ=-0.158, *p*<0.001, γ=-0.23, *p*<0.001). In comparison to males, females with TAO showed a higher proportion of unilateral eye involvement and upper eyelid retraction ≥2mm (*p*=0.038, *p*<0.001). However, males had a higher proportion of exophthalmometry ≥17mm (*p*<0.001). The proportions of patients with eye movement disorder (γ=0.535, *p*<0.001), diplopia (γ=0.446, *p*<0.001), intraocular pressure (IOP) ≥30mmHg (γ=0.149, *p*<0.001), sight-threatening TAO (γ=0.479, *p*<0.001), and active TAO (γ=0.469, *p*<0.001) were positively correlated with age in TAO patients. Additionally, the proportion of male patients is higher than that of female patients.

**Conclusion:**

TAO Patients exhibit different clinical features depending on age and gender. In elderly and male patients, TAO tends to be more severe, with a higher prevalence of eye muscle involvement, IOP ≥30mmHg, active phases, and a lower proportion of patients with upper eyelid retraction ≥2mm. Elderly female patients also have a lower proportion of exophthalmometry ≥17mm.

## Introduction

Thyroid-associated ophthalmopathy (TAO) is an autoimmune orbital inflammatory disease with diverse clinical manifestations (1). TAO patients can range in age from children to the elderly (2). The peak age of onset for males is approximately five years older than females (3, 4). Literature reports a higher incidence of TAO in females compared to males, with a ratio of 6.06-1.76 (2, 5–7). Clinical manifestations and physical signs of TAO patients mainly include eyelid retraction, diplopia, proptosis, and visual impairment, but there can be considerable variation among individuals. Some patients may only experience eyelid retraction or proptosis, while others may have dysthyroid optic neuropathy (DON) caused by the involvement of the extraocular muscles, which seriously impairs the patient’s visual function (1, 8).

Research shows that teenage patients with TAO generally exhibit milder symptoms and rarely experience DON (9, 10), whereas elderly patients commonly suffer from extraocular muscle involvement, with a higher proportion of DON (11). Studies have shown that male TAO patients typically experience more severe symptoms than female patients. However, it has been found that male patients tend to be older than female patients, and age difference may be the main reason for the difference (12). However, previous studies have not controlled for this confounding factor. There have been limited studies on Chinese TAO patients, with only a few research findings reported by foreign studies.

Hence, it is crucial to examine how age and gender affect Chinese patients with TAO in order to provide effective diagnosis and treatment. In this study, TAO patients will be grouped continuously according to age and gender to further clarify the clinical characteristics of TAO patients.

## Method

A single center case series study was conducted to collect data from patients who visited the Orbital Disease Specialist Clinic of West China Hospital, Sichuan University from September 2009 to December 2019. Only patients who had been diagnosed with TAO based on Bartley’s diagnostic criteria (13) and never undergone ocular therapy such as corticosteroid, radiation, and surgical treatment were included. When eyelid retraction is the presenting symptom, the diagnosis of TAO can be established by the presence of one of the following three signs or test results, while excluding other causes: (1) Abnormality in thyroid function or one of the thyroid-related antibodies; (2) Exophthalmos > 14mm, or a difference in exophthalmos between the two eyes of ≥2mm, or progressive exophthalmos; (3) Involvement of extraocular muscles: Orbital CT or orbital MRI showing regular thickening of one or more posterior segments of extraocular muscles without involving the tendons. When abnormality in thyroid function or one of the thyroid-related antibodies is the initial symptom, the diagnosis can be confirmed by the presence of one of the following three signs, while excluding other causes: (1) Eyelid retraction; (2) Exophthalmos; (3) Involvement of extraocular muscles. All patients were diagnosed with TAO and underwent eye evaluations conducted by a senior-level ophthalmologist. The ophthalmologist recorded their medical history, including demographic data, complaints, ocular signs, exophthalmometry, IOP, activity staging (14), severity grading (8) and more. Additionally, the ophthalmologist took facial photos of the patients.

The patients were divided into different age groups as follows: Group 1: ≤18 years old; Group 2: 19-29 years old; Group 3: 30-39 years old; Group 4: 40-49 years old; Group 5: 50-59 years old; Group 6: 60-69 years old; Group 7: ≥70 years old. Additionally, the patients were further divided into male and female groups.

Statistical analyses were conducted using SPSS 25.0 (IBM Corp.). Continuous variables were described as the means and standard deviations (SDs). Categorical variables were shown as numbers and percentages. The Pearson χ2 test was used for two-group and multiple-group categorical variables. When the chi-square test criteria were not met, Fisher’ s exact probability method was used for statistical testing, and *p*<0.05 was considered statistically significant. To address the increased risk of false positives associated with multiple comparisons, we employed the Bonferroni correction method for pairwise comparisons between two groups. Specifically, we divided the predetermined significance level (typically 0.05) by the total number of comparisons conducted to determine the adjusted significance level for each comparison. The Goodman- Kruskal Gamma was used to test the correlation between two categorical variables. The *p*<0.05 was considered statistically significant, and the strength of the association was represented by the γ coefficient.

## Result

A total of 2479 patients with TAO were included in this study, including 854 males (34.45%) and 1625 females (65.55%). The average age of males was 44.95 ± 13.56 years, while it was 41.52 ± 13.03 years for females (*p*<0.001). Group 1 and Group 7 had a small combined proportion of 2.70% and 2.10%, respectively. In contrast, Group 4 had the largest proportion of 29.65%. In Group 1, the female to male patient ratio was 3.79, while in Group 7, it was only 0.86. The proportion of females decreased gradually with increasing age (γ=-0.168, *p*<0.001), as shown in **Table 1**. The female-to-male ratio showed statistically significant differences between Group 1/4 and Group 6/7, Group 2 and Groups 5/6/7, and Group 3 and Group 6 (*p*<0.001).

**Table 1:** Demographic of 2479 TAO patients.

Age categories age (years)	Age (years, average±SD)	Patient number (%)	Male number (%)	Female number (%)	Female: Male
Group 1: age ≤18	14.75±3.11	67 (2.70)	14 (1.64)	53 (3.26)	3.79a,b
Group 2: age 19-29	25.19±2.91	386 (15.57)	109 (12.76)	277 (17.05)	2.54b
Group 3: age 30-39	34.31±2.88	529 (21.34)	175 (20.49)	354 (21.78)	2.02a,b,c
Group 4: age 40-49	44.59±2.75	735 (29.65)	235 (27.52)	500 (30.77)	2.13a,b
Group 5: age 50-59	53.63±2.82	475 (19.16)	183 (21.43)	292 (17.97)	1.6a,c,d
Group 6: age 60-69	63.35±2.68	235 (9.48)	110 (12.88)	125 (7.69)	1.14d
Group 7: age ≥70	74.15±3.76	52 (2.10)	28 (3.28)	24 (1.48)	0.86c,d

Each group in Female: Male column is labeled with different letters, such as a, b, c, d. When two groups do not have the same letter, it indicates a significant statistical difference between them; When there is a shared letter between two groups, it indicates that there is no statistical difference between the two groups.

**Figure 1:**
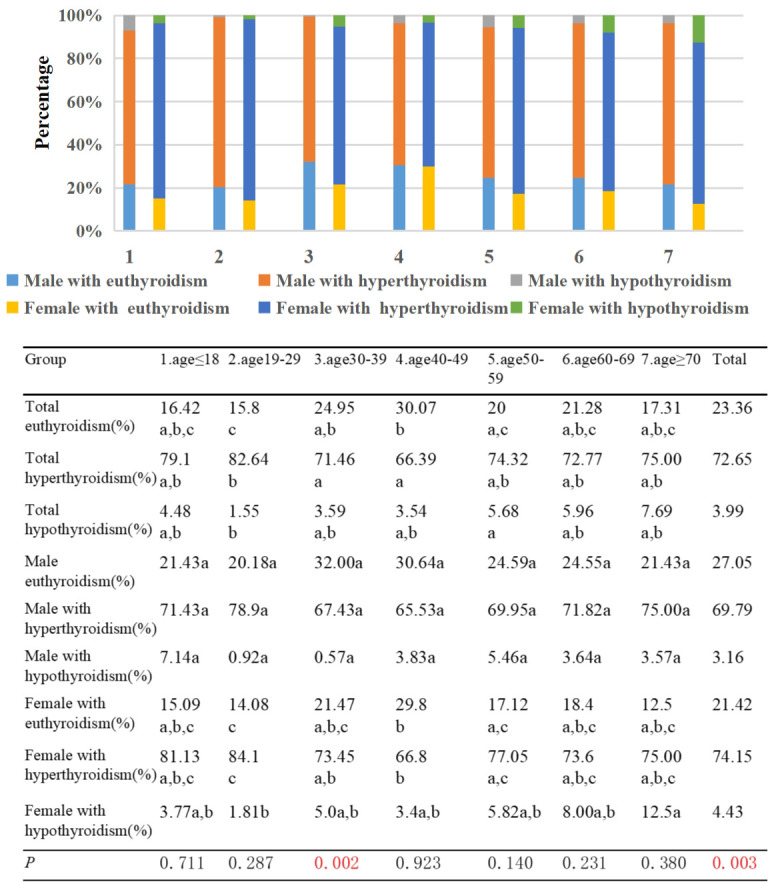
Percentage of TAO patients with euthyroid, hyperthyroidism, and hypothyroidism by different age groups and genders. Each group in different thyroid functions is labeled with different letters, such as a, b, c. When two groups do not have the same letter, it indicates a significant statistical difference between them; When there is a shared letter between two groups, it indicates that there is no statistical difference between the two groups.

The highest percentage of patients with euthyroidism was observed in Group 4, accounting for 30.07% of the TAO patients. There was a statistically significant difference in the proportion of TAO patients with euthyroidism between Group 2 and Groups 3/4, as well as between Groups 3 and 4 (*p*<0.01). In Group 2, the proportion of patients with hyperthyroidism was the highest, reaching 82.64%, and this proportion differed significantly from that of Groups 3 and 4 (*p*<0.01). Furthermore, the proportion of male patients with euthyroidism was significantly higher than that of female patients (*p*=0.003). Although the number of male patients with euthyroidism was higher than that of female patients in each age group, this difference reached statistical significance only in Group 3 (*p*=0.002) (**Figure 1**).

**Figure 2:**
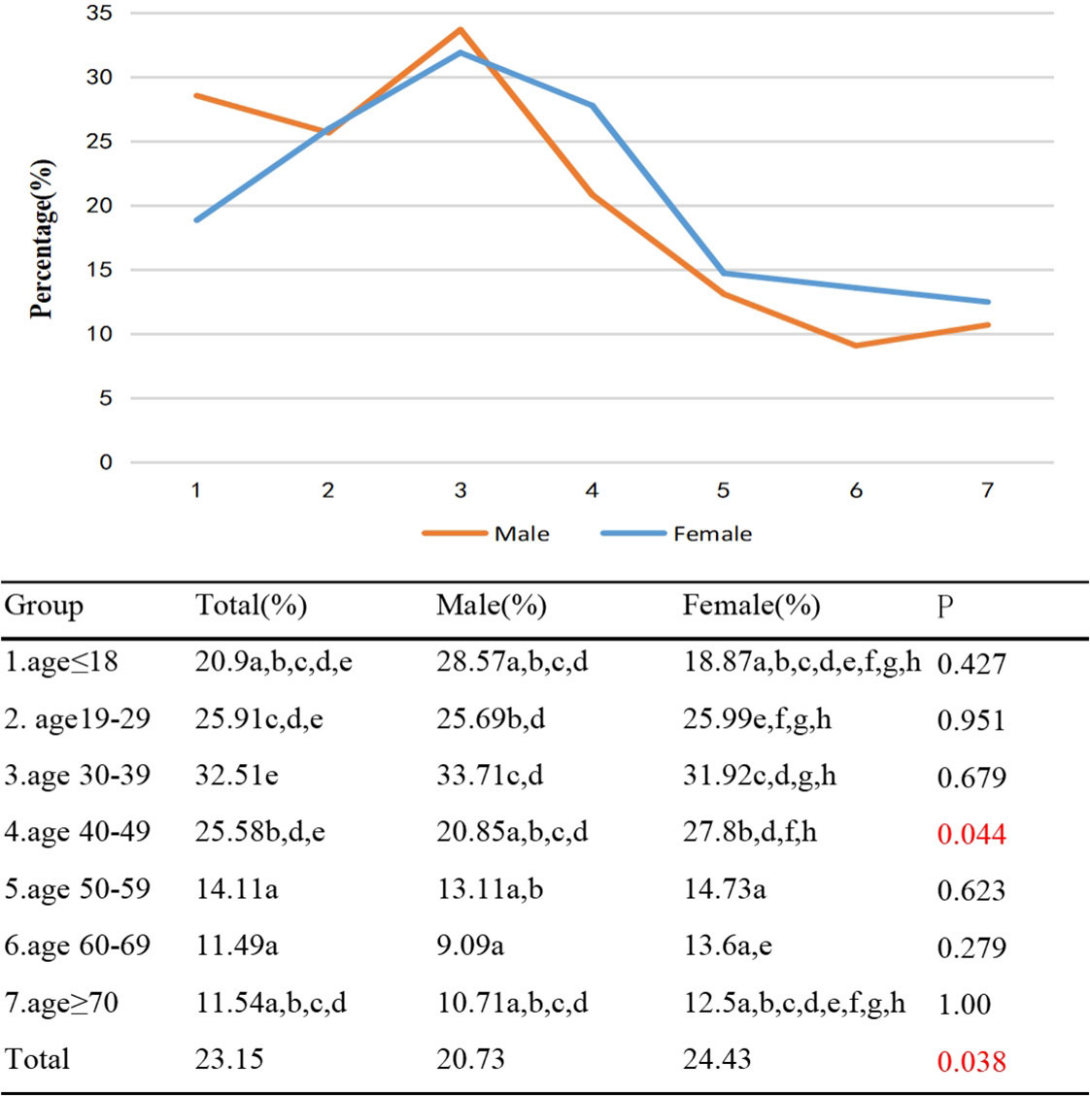
Percentage of TAO patients with unilateral involvement by different age groups and genders. Each group in each column is labeled with different letters, such as a, b, c, d, e, f, g, h. When two groups do not have the same letter, it indicates a significant statistical difference between them; When there is a shared letter between two groups, it indicates that there is no statistical difference between the two groups.

The highest proportion of patients with unilateral involvement in TAO was observed in Group 3, reaching 32.51%. As age increased, the proportion of patients with unilateral involvement decreased significantly (γ=-0.23, *p*<0.001). There was a significant difference in the proportion of TAO patients with unilateral involvement between Groups 2/3/4 and Groups 5/6. Additionally, the proportion of females with unilateral involvement in TAO was 24.43%, while for males it was 20.73% (*p*=0.038) (**Figure 2**).

**Figure 3:**
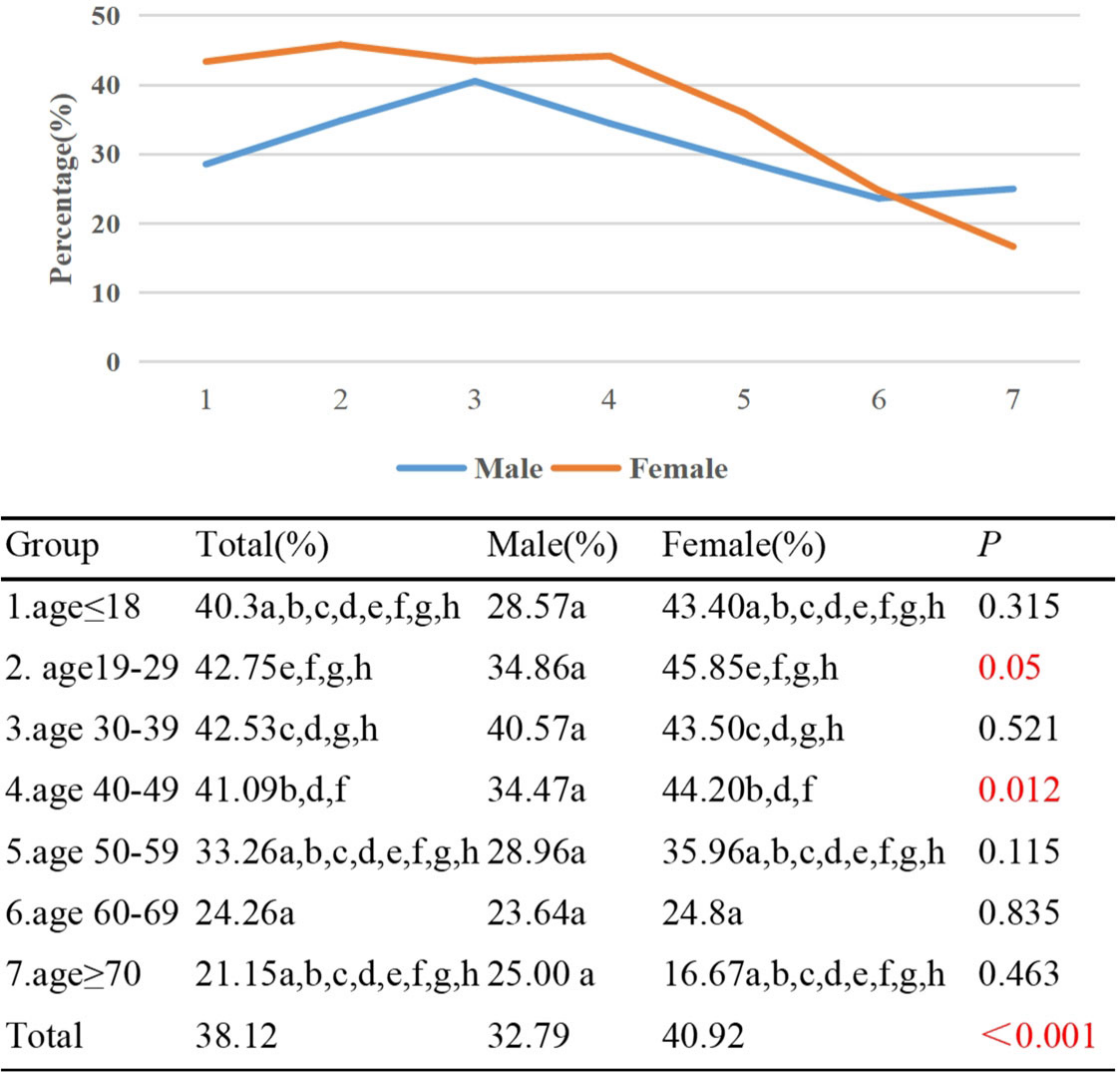
Percentage of TAO patients with upper eyelid retraction ≥2mm by different age groups and genders. Each group in each column is labeled with different letters, such as a, b, c, d, e, f, g, h. When two groups do not have the same letter, it indicates a significant statistical difference between them; When there is a shared letter between two groups, it indicates that there is no statistical difference between the two groups.

In patients with TAO, the prevalence of upper eyelid retraction ≥2mm was found to be approximately 40% in Group 1, Group 2, Group 3, and Group 4 respectively. In contrast, the prevalence was lower at 21.15% in Group 7. This study also observed a negative correlation between age and the prevalence of upper eyelid retraction ≥2mm (γ=-0.158, *p*<0.001). Furthermore, a statistically significant difference was observed in the prevalence of upper eyelid retraction ≥2mm between Groups 2/3/4, and 6. Among females, the prevalence of upper eyelid retraction ≥2mm was 40.92%, while among males it was 32.79% (*p*<0.001). Except for Group 7, the proportion of females with upper eyelid retraction ≥2mm was higher than that of males in each age group, with statistically significant differences observed only in Group 2 and Group 4 (*p*<0.05) (**Figure 3**).

**Figure 4:**
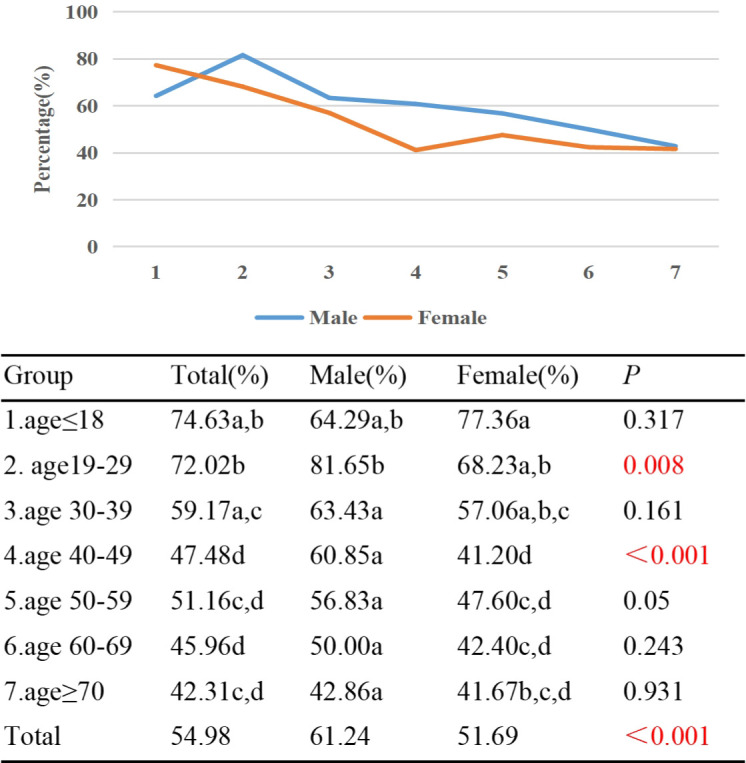
Percentage of TAO patients with exophthalmometry≥17mm by different age groups and genders. Each group in each column is labeled with different letters such as a, b, c, d. When two groups do not have the same letter, it indicates a significant statistical difference between them; When there is a shared letter between two groups, it indicates that there is no statistical difference between the two groups.

The study found that Group 1 had the highest percentage (74.63%) of TAO individuals with exophthalmometry ≥17mm, while Group 7 had a lower proportion of 42.31%. Additionally, there was a negative correlation between the proportion of individuals with exophthalmometry ≥17mm and age (γ=-0.233, *p*<0.001). Statistical differences in the proportions of individuals with exophthalmometry ≥17mm were observed between Groups 1/2 and Groups 4/5/6/7, as well as between Group 3 and Groups 2/4/6. In terms of gender, 51.69% of females had exophthalmometry measurement ≥17mm, while 61.24% of males had the same measurement (*p*<0.001). Except for Group 1, the proportion of males with exophthalmometry ≥17mm was higher than that of females in all other age groups, with statistical differences observed in Group 2 and Group 4 (**Figure 4**).

**Figure 5:**
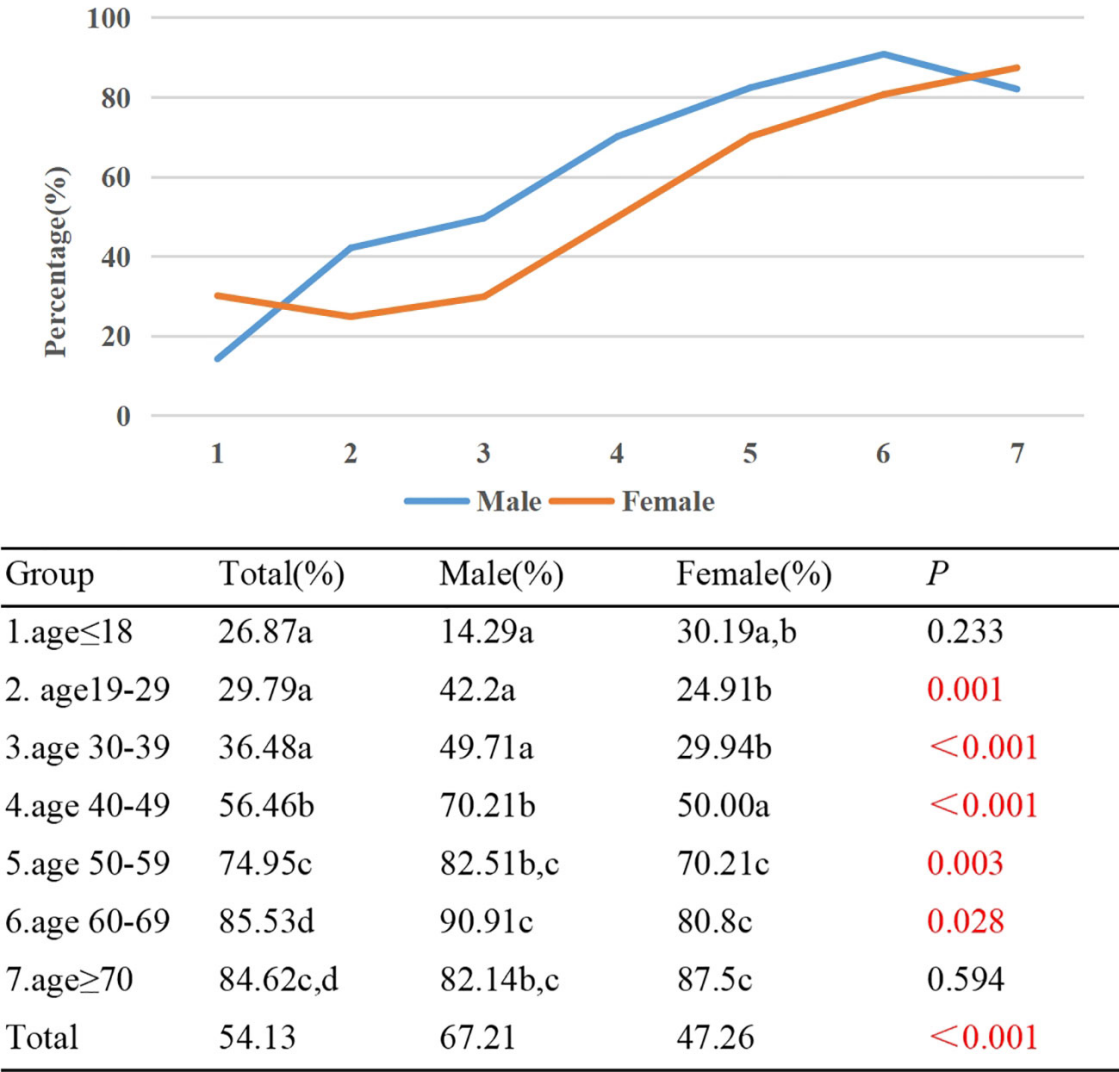
Percentage of TAO patients with eye movement disorder by different age groups and genders. Each group in each column is labeled with different letters, such as a, b, c, d. When two groups do not have the same letter, it indicates a significant statistical difference between them; When there is a shared letter between two groups, it indicates that there is no statistical difference between the two groups.

In Group 1, only 26.8% of patients with TAO displayed eye movement disorder. However, in Groups 6 and 7, the percentages rose significantly to 85.53% and 84.62% respectively. Age was found to have a positive correlation with the proportion of TAO patients experiencing eye movement disorder (γ=0.535, *p*<0.001). Notably, statistically significant differences in the proportions of eye movement disorder were observed between Group 1/2/3, and Group 4/5/6/7. Additionally, significant differences were found between Group 4 and Group 5/6/7, as well as between Group 5 and Group 6. Among the participants, 67.21% of males exhibited eye movement disorder, in contrast to 47.26% of females (*p*<0.001). Except for Group 1 and Group 7, the proportion of male patients with eye movement disorder exceeded that of female patients in all other age groups, and these differences were statistically significant (*p*<0.05) (**Figure 5**).

**Figure 6:**
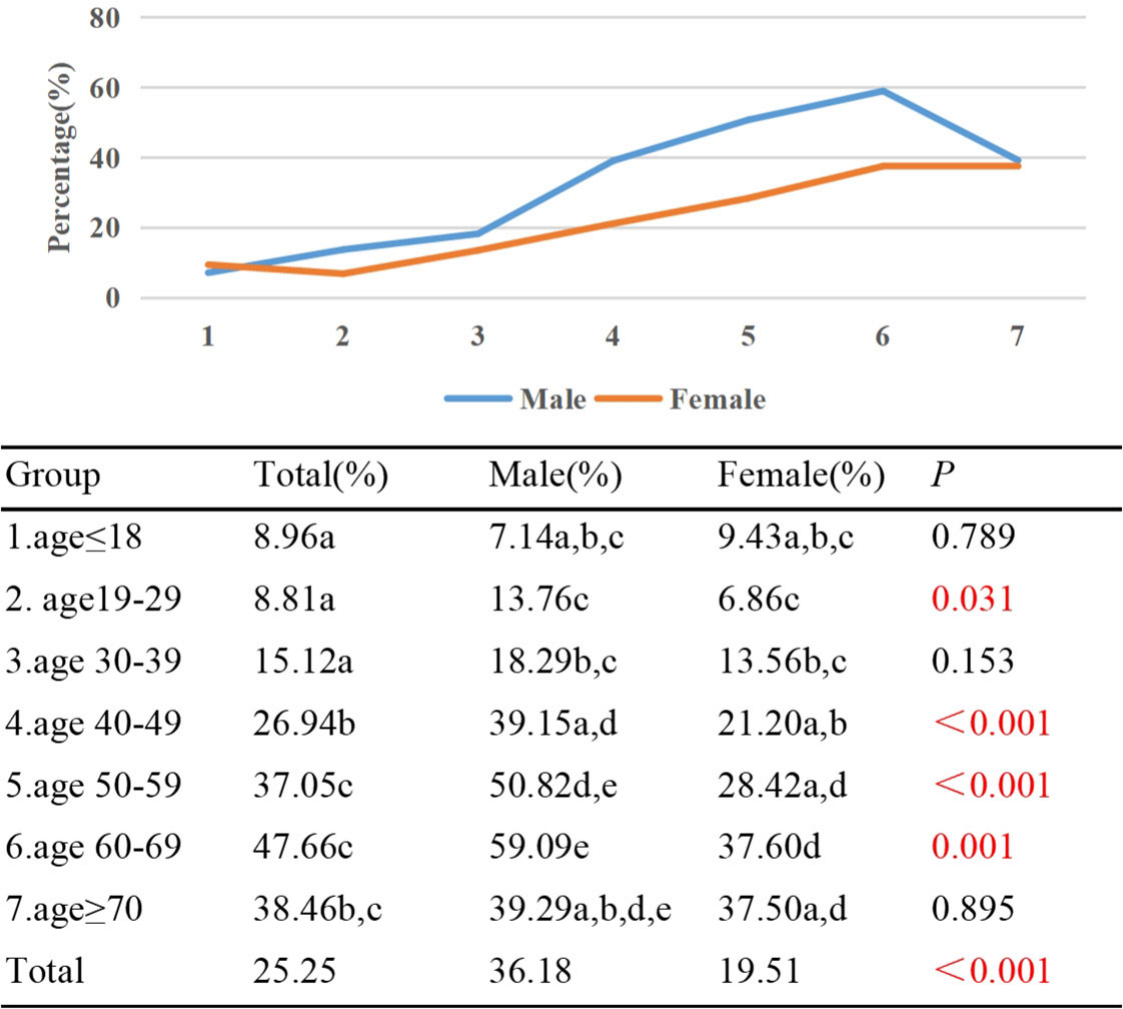
Percentage of TAO patients with diplopia by different age groups and genders. Each group in each column is labeled with different letters, such as a, b, c, d, e. When two groups do not have the same letter, it indicates a significant statistical difference between them; When there is a shared letter between two groups, it indicates that there is no statistical difference between the two groups.

In Group 1, only 8.96% of TAO patients experienced diplopia, while the highest prevalence of diplopia, at 47.66%, was observed in Group 6. An upward trend was observed in the proportion of diplopia with increasing age (γ=0.446, *p*<0.001). Significant variations in the diplopia proportion were noted between Group 1/2/3 and Group 4/5/6/7. Additionally, differences were identified between groups 4 and 5, as well as 4 and 6. A gender-based analysis indicated a diplopia prevalence of 36.18% among males and 19.51% among females (*p*<0.001). Across all age groups except for Group 1, a higher proportion of males experienced diplopia compared to females, with statistically significant differences observed in Group 2, 4, 5, and 6 (*p*<0.05) (**Figure 6**).

**Figure 7:**
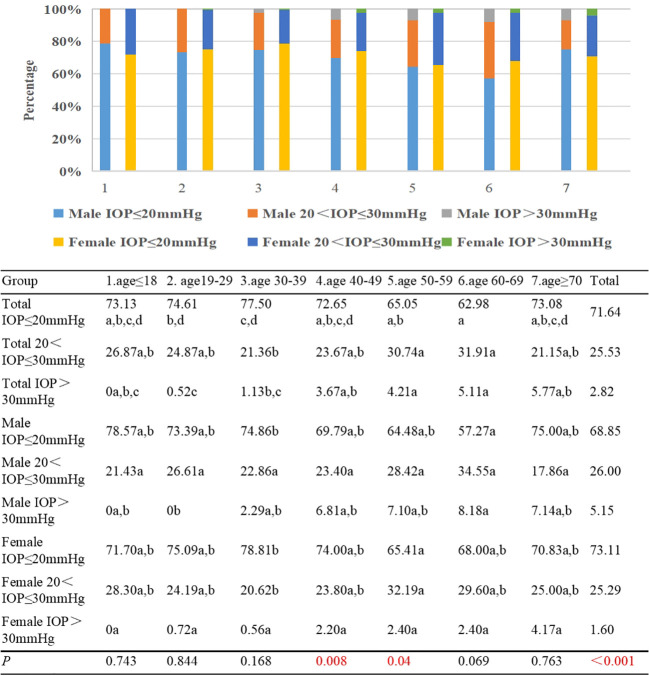
Percentages of TAO patients with IOP ≤20mmHg, 20 <IOP ≤30mmHg, and >30mmHg by different age groups and genders. Each group in each row is labeled with different letters, such as a, b, c, d. When two groups do not have the same letter, it indicates a significant statistical difference between them; When there is a shared letter between two groups, it indicates that there is no statistical difference between the two groups.

In Group 1, no cases of patients with TAO exhibited an IOP ≥30mmHg, whereas in Group 7, this proportion reached 5.77%. Notably, the incidence of elevated IOP ≥30mmHg displayed a gradual increment with advancing age (γ=0.149, *p*<0.001). Comparisons between Group 2 and Groups 3/4/5/6 revealed significant statistical disparities concerning the prevalence of IOP ≥30mmHg, as well as between Group 3 and Groups 5/6. Stratified by gender, the prevalence of IOP ≥30mmHg was 5.15% in male patients and 1.6% in their female counterparts (*p*<0.001). Furthermore, across each age subgroup, the proportion of males exhibiting IOP ≥30mmHg exceeded that of females, a distinction that attained statistical significance in Group 4 and Group 5 (*p*=0.008, *p*=0.04) (**Figure 7**).

**Figure 8:**
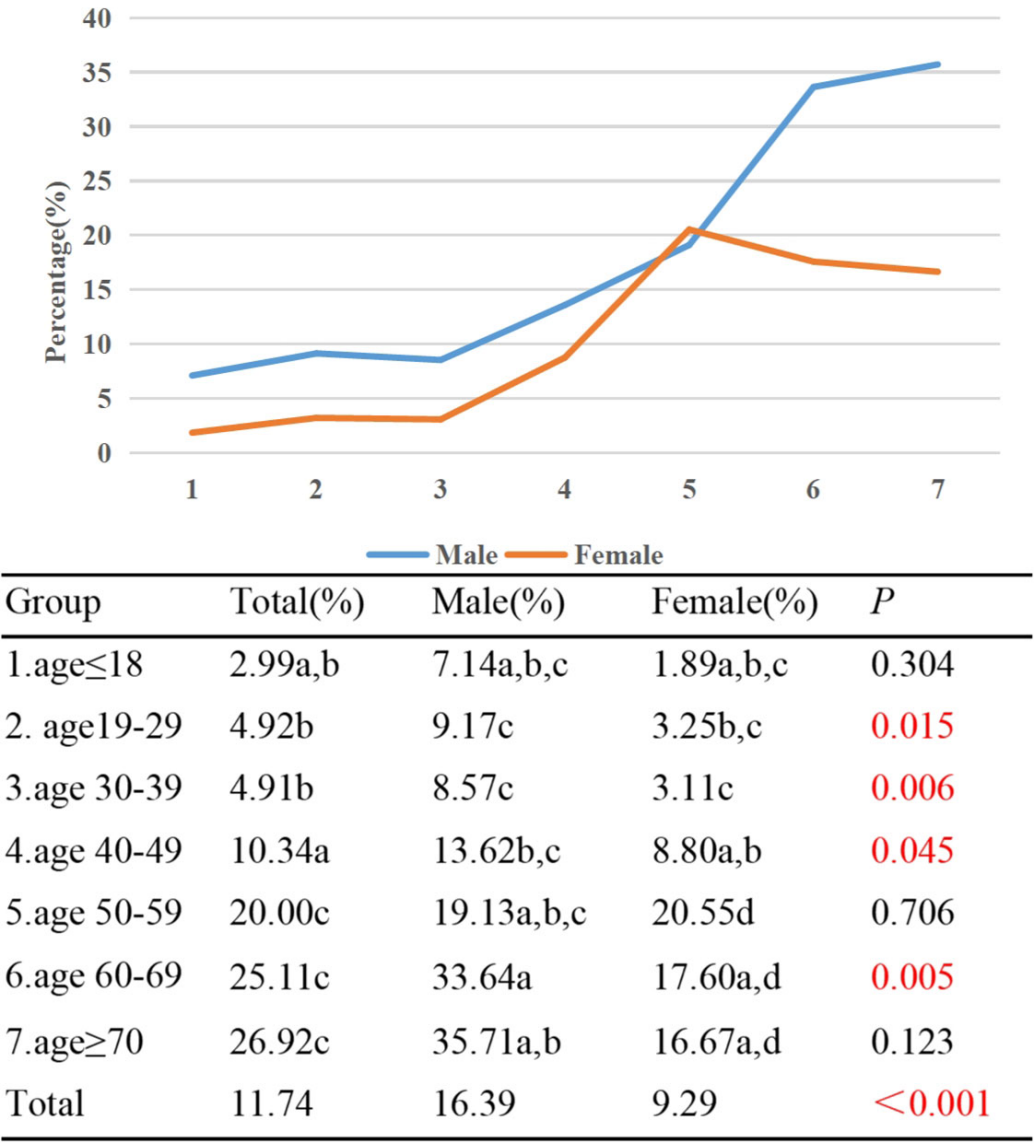
Percentages of active TAO patients by different age groups and genders. Each group in each column is labeled with a different letter, such as a, b, c, d. When two groups do not have the same letter, it indicates a significant statistical difference between them; When there is a shared letter between two groups, it indicates that there is no statistical difference between the two groups.

In Group 1, the percentage of active TAO patients was 2.99%, contrasting with Group 7 where this figure reached 26.92%. Notably, an upward trend was observed as age advanced, with a positive correlation between age and the incidence of active TAO (γ=0.469, *p*<0.001). Significantly divergent proportions of active TAO patients were evident when comparing Group 1/2/3/4 against Group 5/6/7; additionally, a statistically significant discrepancy emerged between Group 2/3 and Group 4. Active male TAO patients accounted for 16.39% of cases, in contrast to 9.29% among females (*p*<0.001). With the exception of Group 5, a consistent pattern emerged across all other age groups, where the prevalence of active TAO was higher among males compared to females, and this difference was statistically significant in Group 2/3/4/6 (*p*<0.05) (**Figure 8**).

**Figure 9:**
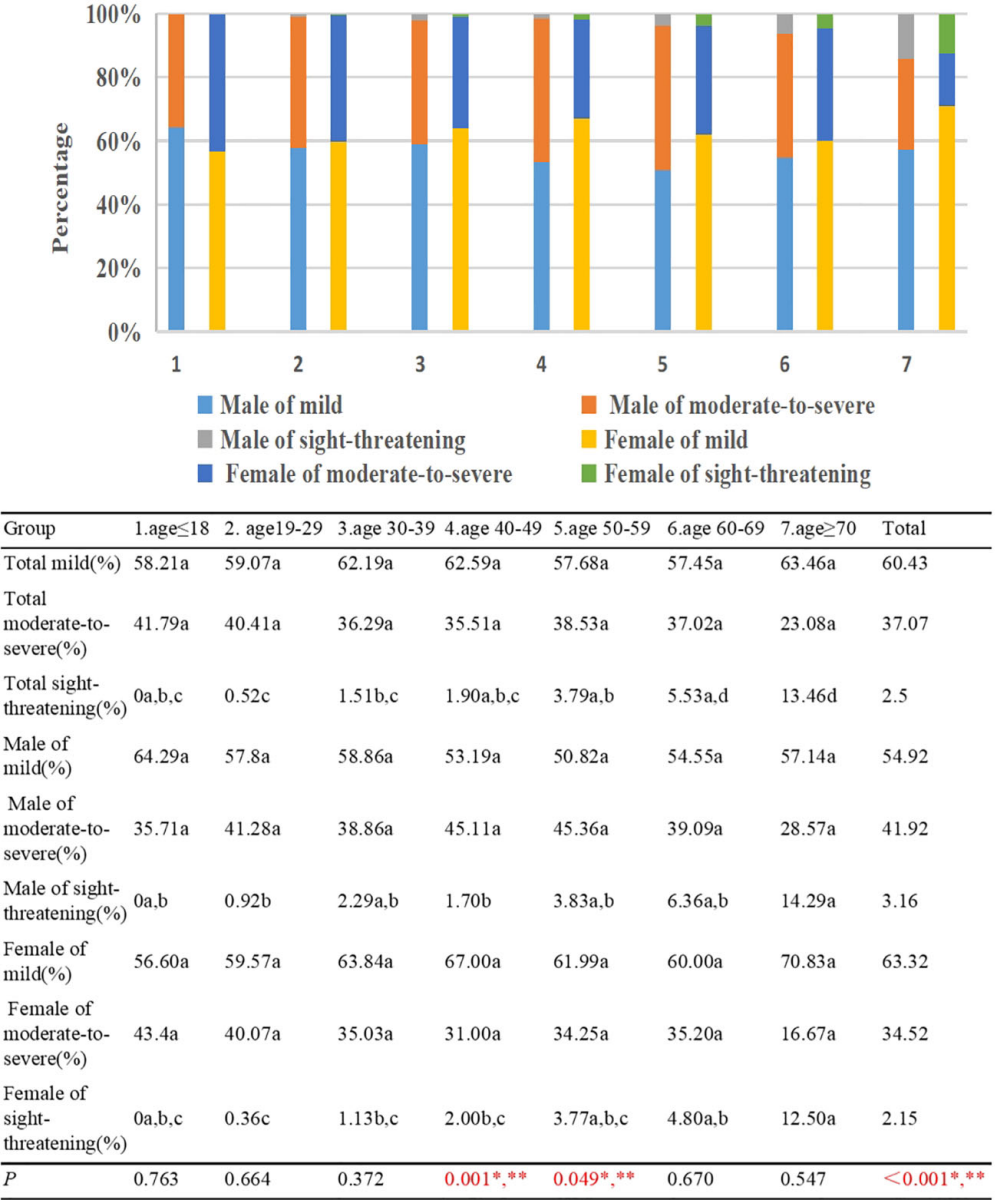
Percentage of mild, moderate to severe, sight-threatening TAO patients by different age groups and gender. Each group in each row is labeled with a different letter, such as a, b, c, d. When two groups do not have the same letter, it indicates a significant statistical difference between them; When there is a shared letter between two groups, it indicates that there is no statistical difference between the two groups.*There is a statistically significant difference in the proportion of mild TAO patients between males and females, **There is a statistically significant difference in the proportion of moderate to severe TAO patients between males and females.

No patients of sight-threatening TAO were identified within Group 1. However, within Group 7, a notable 13.46% of patients exhibited sight-threatening TAO. Upon stratifying the severity of TAO into two categories, specifically those at risk of sight-threatening complications and those without such risks, a discernible trend emerged. This trend demonstrated an incremental rise in the prevalence of sight-threatening TAO with advancing age (γ=0.497, *p*<0.001). Noteworthy distinctions in the occurrence of sight-threatening TAO were observed among Group 1/2/3/4/5 and Group 7. Moreover, statistically significant differences were noted between Group 2 and Groups 5/6, as well as between Group 3 and Group 6. In terms of gender disparities, mild TAO cases accounted for 54.92% among males and 63.32% among females, while patients with moderate to severe TAO constituted 41.92% among males and 34.52% among females (*p*<0.001) (**Figure 9**). 

## Discussion

In this investigation, an analysis of the clinical attributes among patients with TAO was undertaken, employing systematic categorization by age and gender. The findings revealed discernible variations in clinical traits across diverse age groups and genders among TAO patients. Notably, the age range of 40-49 years old exhibited the highest representation, constituting 29.65% of the cohort, while the ≤18 years old group displayed the lowest prevalence at 2.70%. It is important to emphasize that TAO exhibits a notable connection with hyperthyroidism. The relatively diminished representation of adolescents within the TAO patient population might be attributed to the lower incidence of hyperthyroidism observed in this age group (15).

While numerous research reports have addressed regional and racial disparities in the gender distribution among patients with TAO, it is widely acknowledged that a notable predominance of female patients exists (2). Our investigation revealed a female-to-male ratio of 3.79 among individuals aged ≤18 years old, contrasting with a ratio of 0.86 for those aged ≥70 years old. Notably, there was an inverse correlation between age and the proportion of female patients. A separate study conducted in Iran indicated that the female-to-male ratio for TAO patients was 1.6 for individuals <29 years, 2.25 for 30-49 years, and 0.6 for individuals aged 50-70 years (16). Similarly, a study in Israel identified a female-to-male ratio of 0.73 for TAO patients aged ≥80 years (17). However, the underlying factors contributing to these variations necessitate further comprehensive investigation.

The prevalence of unilateral TAO in the literature varied for 9-36% (2, 18). This current investigation examines the distribution of unilateral TAO across diverse age cohorts, revealing a range of 11.49% to 32.51%. A discernible inverse relationship between age and the incidence of unilateral TAO becomes apparent, with a higher occurrence rate observed among females in contrast to their male counterparts. Consistent with our study’s findings, research conducted in Iran underscores that unilateral TAO primarily affects younger individuals, predominantly females, as opposed to cases of bilateral TAO (19). Additionally, an investigation from Israel underscores that bilateral TAO was notably prevalent among patients aged ≥80 years in contrast to the younger age brackets (i.e., <40 years and 41-79 years) (17). Notably, a multicenter study conducted in the United Kingdom corroborated that female constituted a higher proportion within the unilateral TAO category compared to the bilateral TAO category (20).

Proptosis and eyelid retraction were primary clinical features observed in patients diagnosed with TAO (21). The findings of this study revealed a negative association between the age of TAO patients and the percentage of individuals exhibiting exophthalmometry ≥17mm and upper eyelid retraction ≥2mm. Specifically, a greater proportion of female patients displayed upper eyelid retraction ≥2mm, while a higher percentage of male patients demonstrated exophthalmometry ≥17mm. A research conducted in Japan involving 10,931 TAO patients similarly illustrated a negative correlation between age and proptosis (γ=-0.312), with male patients exhibiting greater proptosis compared to their female counterparts. Moreover, patients with eyelid retraction tended to be younger, and the proportion of female patients affected was higher (4). Additionally, a multi-center research conducted across the Netherlands and Iran included a sample of 115 patients aged below <18 years, revealing a higher prevalence of exophthalmos and eyelid retraction in these young patients compared to adult individuals diagnosed with TAO (10).

Our research has illuminated a noteworthy positive correlation between the age of patients afflicted by TAO and the incidence of both eye movement disorders and diplopia. Additionally, this pattern is accentuated by a higher representation of male individuals within this cohort. These trends align with the observations put forth by Simon et al., who documented a diplopia incidence of 17% among TAO patients aged ≤40 years, contrasting with a substantially elevated 54% among those aged >40 years (22). The investigation conducted by Regensburg et al. has introduced discrete classifications for TAO patients based on orbital CT scans. Strikingly, individuals characterized by heightened proliferation of orbital adipose tissue exhibited a younger age demographic and more pronounced proptosis. In contrast, patients exhibiting involvement of the extraocular muscles tended to be older and displayed an increased predisposition to diplopia (23). However, a comprehensive grasp of the precise pathogenic mechanisms that underlie these clinical variations necessitates further comprehensive inquiry.

A meta-analysis of 56 included articles revealed a significant positive correlation between male gender (CI: 1.178-2.808) and age (CI: 3.075-8.017) with active TAO (24). Our study also demonstrated that age positively correlates with the proportion of TAO patients in the active stage. Furthermore, there was a significantly higher proportion of male patients in the active stage compared to female patients.

A study conducted in the UK suggested a positive correlation between age and the severity of TAO, with male patients experiencing more severe symptoms compared to female patients (12). Similarly, a retrospective study in Germany involving 4260 TAO patients showed a higher proportion of moderate to severe and sight-threatening cases in patients aged ≥50years, particularly among males (11). In our research, we utilized the TAO severity grading system developed by the EUGOGO. Our findings indicated that there was no statistically significant difference in the composition ratio of mild and moderate-to-severe cases across different age groups. However, we did observe a positive correlation between the proportion of sight-threatening TAO cases and age. Furthermore, the proportion of male patients with moderate-to-severe cases was higher than that of female patients. This might be attributed to a higher occurrence of extraocular muscle involvement among elderly patients, leading to thickening of the extraocular muscles and compression of the optic nerve, resulting in DON.

**Figure 10:**
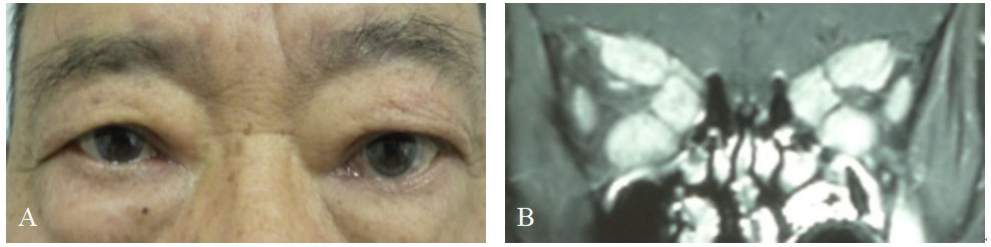
Representative examples of thyroid-associated ophthalmopathy (TAO) patients only with extraocular muscles involvement. **(A)** TAO patients without upper eyelid retraction and conjunctival congestion, chemosis, and prominent eyes. **(B)** Coronal enhanced MRI scans showing thickening and infammation of the superior, inferior and medial rectus muscle of the bilateral eye.

Previous studies have suggested that males are less sensitive to changes in their appearance compared to females and have a poorer adherence to treatment for hyperthyroidism (25). This may explain why male patients often have more severe symptoms when seeking medical attention. However, further research is needed to explore other influencing factors, such as the impact of gender-related hormone levels. In some cases, elderly TAO patients might not exhibit noticeable symptoms like eyelid retraction and proptosis, despite severe involvement of the extraocular muscles detected through orbital imaging (**Figure 10**). According to the EUGOGO classification system, they can only be graded as mild. Moreover, the EUGOGO grading system for assessing the severity of TAO still has some shortcomings that require improvement. To enhance its efficacy, adding orbital imaging examination results to the grading system could offer greater guidance for diagnosis and treatment.

Our research has certain limitations. This study is based on data collected from a national tertiary care center specializing in complex and severe cases in the southwestern region of China. Patients were recruited from various provinces in western China and other regions, which may introduce some degree of regional bias. Given that the onset age of TAO is mainly between 40-69 years old (2), it is worth noting that the sample size in our study for the age groups of ≤ 18 and ≥ 70 years old is relatively limited, which may introduce bias. Nevertheless, inclusion of these age groups is essential as they represent the adolescent and elderly demographics. Adjustments have been made to address this issue in the statistical analysis. Previous studies have indicated that smoking and thyroid function can impact the severity of TAO. Not controlling for smoking and thyroid function, among other confounding factors, may introduce bias into the study results. The primary aim of this study is to investigate the clinical manifestations of TAO in different age and gender groups, aiming to assist clinicians in identifying distinct TAO characteristics in various populations for expedited diagnosis. Therefore, we aim to focus on the influence of these two factors on the outcomes to ensure a clear and precise research focus. Future research could delve deeper into the effects of smoking and thyroid function status, along with incorporating additional factors for a comprehensive analysis, to enhance the breadth and credibility of research conclusions.

Nevertheless, the population dataset we examined is sufficiently large to draw reasonable conclusions. While our research observed clinical performance variations among TAO patients of different genders and ages, further investigation is needed to understand the underlying reasons for these differences. For instance, variations in hormone levels between different age groups and genders may potentially impact the clinical presentation of TAO.

## Conclusion

The study reveals that patients with TAO exhibit distinct clinical characteristics based on age and gender. Elderly and male patients demonstrate a higher prevalence of eye movement disorder, diplopia, and sight-threatening TAO. Conversely, a greater proportion of young patients experience upper eyelid retraction ≥2mm and exophthalmometry ≥17mm. Healthcare practitioners involved in the diagnosis and treatment of TAO should conduct more rigorous assessments and implement early therapeutic measures, particularly for elderly and male patients. Understanding the clinical manifestations of TAO patients across different age and gender groups can aid clinicians in better identifying patients, accurately predicting prognosis, and tailoring personalized treatments.

## Data Availability

The original contributions presented in the study are publicly available. This data can be found here: https://www.jianguoyun.com/p/DXA-Y5wQ0aD2DBijqNcFIAA.
